# Lung POCUS in a Pulmonary Outpatient Clinic: Balancing Utility and Feasibility

**DOI:** 10.24908/pocusj.v10i02.19661

**Published:** 2025-11-17

**Authors:** Priyanka Sridhar, Paru Patrawalla, Boram Kim

**Affiliations:** 1 Division of Pulmonary, Critical Care and Sleep Medicine, Department of Medicine, Mount Sinai Morningside and West, Icahn School of Medicine at Mount Sinai, New York City, NY 10019, USA.

**Keywords:** Point of Care Ultrasonography, Lung POCUS, Pleural ultrasound, Outpatient POCUS, Pulmonary clinic

## Abstract

**Background:**

Despite abundant literature supporting the diagnostic utility of lung point of care ultrasound (POCUS) in inpatient settings, there is limited data on the feasibility and utility of lung POCUS in pulmonary outpatient clinics. Patients may first seek care for dyspnea in a pulmonary outpatient clinic. Existing data on in-hospital use suggests that integrating lung POCUS into clinic visits may lead to earlier diagnosis and limit the need for additional testing [1,2].

**Objective:**

The aim of this observational study was to evaluate the feasibility, clinical impact, indications, practices, and findings associated with lung POCUS in an urban-based pulmonary outpatient clinic.

**Methods:**

We reviewed 100 consecutive patients who underwent a lung POCUS exam during their pulmonary outpatient clinic visit. Lung POCUS was performed by trainees and faculty, stored in a cloud-based archival system, and reviewed by pulmonary attendings. Findings were categorized into three patterns: predominant A-line, predominant B-lines, and predominant A-lines with a B-line focus. Summary statistics were performed using the data.

**Results:**

The mean age of the included patients was 57+/- 17 years, and 64% were female. Residents performed 70% of the studies. The median time for a lung POCUS exam was 5 minutes. A normal lung POCUS with a predominant bilateral A-line pattern (71%) was most commonly associated with obstructive airway disease (31%). A bilateral B-line pattern (13%) was associated with either interstitial lung disease (7%) or heart failure (8%). Focal dense B-lines (10%) were seen with atelectasis (3%) or other abnormal computed tomography (CT) findings (3%). Lung POCUS demonstrated stability versus progression of pleural effusions in 18% of cases. Of the 49% of patients who had additional imaging ordered for them, there was 100% concordance between lung POCUS findings and chest X-ray (CXR). We explored the potential impact of lung POCUS on clinical management in five cases.

**Conclusions:**

Lung POCUS is feasible to perform in a pulmonary outpatient clinic without adding a significant amount of time to the patient encounter. There is strong concordance between a normal lung POCUS and CXR, which can supplant the need for CXRs in certain conditions. Areas of future research include evaluating providers' attitudes towards lung POCUS use in the clinic setting and integrating lung POCUS into clinic workflow.

## Introduction

Point of care ultrasound (POCUS) is well accepted as a diagnostic tool by many providers in the emergency department (ED) and inpatient setting, including the intensive care unit (ICU). The ability to provide real-time information without exposing patients to harmful radiation, unlike other commonly used imaging modalities such as chest X-ray (CXR) and computed tomography (CT), is one of the major reasons for its favorable acceptance among diverse specialties. Despite its widespread use in hospitals and EDs, its role in outpatient settings, specifically in pulmonary outpatient clinics, is not as well understood.

In the ED, lung POCUS has been associated with reduced time to diagnosis for dyspnea and faster administration of disease-specific treatment in patients experiencing exacerbations of chronic obstructive pulmonary disease (COPD) or heart failure [3,4]. When evaluating patients presenting to the ED with undifferentiated chest pain and shortness of breath, lung POCUS demonstrated higher specificity than CXR for detecting pneumothorax and pleural effusion, and helped to narrow the differential diagnosis [5].

There are fewer reported cases of lung POCUS in the outpatient clinic setting. In a study conducted among general practitioners using POCUS in the evaluation of clinic patients, POCUS led to a change in diagnosis in 49.4% patients, a change in management in 50.9%, and a reduction in referrals from 49.2% to 25.6% [6]. Specifically, when looking at heart and lung POCUS, an overall change in management (including consultations, referrals to hospitals for additional imaging, and plans for follow-up visits) occurred in 85% and 84%, respectively [6]. Similarly, Avriel et al. showed that in an ambulatory pulmonary hypertension clinic, POCUS use resulted in significantly more diagnostic and treatment changes when compared to the controls. This association remained even when studied in a multivariate analysis with age, sex, body mass index and symptoms as variables [7].

All pulmonary physicians at our outpatient practice are trained in lung POCUS and are eligible to bill for its use. However, we did not previously have data on current trends of lung POCUS use within our outpatient practice. To address this, we conducted an internal survey of pulmonary faculty and fellows. Of the respondents, 87.5% reported that they believe lung POCUS is useful in the ambulatory setting, and 75% of the respondents indicated that they have used lung POCUS in the pulmonary outpatient clinic. All respondents identified time constraints as the primary barrier to lung POCUS use. Time is a common barrier for lung POCUS use, and has been reported as such in surveys of faculty and trainees working in different inpatient and outpatient settings [8–11]. Due to interest expressed by faculty and fellows in incorporating a streamlined system for lung POCUS into our outpatient clinical practice, we were able to justify a request to the pulmonary division for two handheld machines for use in our pulmonary clinic. Functional workflows to quantify time and other barriers to lung POCUS use in outpatient settings have not been widely studied. Therefore, we conducted a retrospective review of 100 consecutive patients who underwent lung POCUS during their pulmonary outpatient clinic visit to evaluate its potential impact on patient management and clinical workflow.

## Methods

### Study design and population

This was a single center retrospective observational study of consecutively collected data, conducted at the Respiratory Institute at Mount Sinai Downtown in New York City. Pulmonary patients presenting to the Mount Sinai Downtown Respiratory Institute are scheduled to see board-certified pulmonary faculty or Pulmonary and Critical Care Medicine (PCCM) fellows. Rotating internal medicine residents and PCCM fellows evaluate patients under the supervision of pulmonary faculty. Between January 11, 2023 and October 15, 2023, we reviewed the first 100 patients who had a lung POCUS examination during their pulmonary outpatient clinic visit. Patient charts were reviewed for demographic information, co-morbid conditions, reason for clinic visit, findings at the clinic visit, tests ordered, and final diagnosis.

This project was deemed a quality improvement (QI) project by the QI Committee in the Department of Medicine at our institution. The project was also reviewed by the Institutional Review Board (IRB), which granted a Human Research Exempt Determination status. The approval documentation from both the QI Committee and IRB has been attached separately.

### Patient and public involvement

Between January 11, 2023 and October 15, 2023, 100 patients presented to the pulmonary outpatient clinic on routine visits and had lung POCUS performed as part of a physical exam. An IRB review granted our research a Human Research Exempt Determination status since all information collected was stored in a de-identified QI database with no protected health information present, for retrospective review by the study researchers. In this pilot QI study, patients were not involved in the study design, conduct, or addressing the research question of feasibility and utility of lung POCUS in an outpatient pulmonary clinic.

### Lung point of care ultrasound examinations

Attendings, fellows, and residents trained in POCUS were informed that ultrasound machines were available in the clinic and encouraged to save all POCUS images to a cloud-based archival system (QpathE, Telexy Healthcare Inc, British Columbia, Canada). All images captured by a resident or fellow were reviewed by trained pulmonology attendings within 24-48 hours. POCUS exams performed from January 11, 2023 to October 15, 2023 were included in this study. Lung POCUS was performed using the handheld Lumify (Philips Healthcare, Cambridge, MA). The BLUE protocol was adopted for our outpatient pulmonary practice [12]. At least six points were scanned for lung POCUS, but more lung zones were included at the provider's discretion. Physicians may have additionally performed a cardiac or lower extremity POCUS depending on the case. POCUS findings and the total time for each exam were recorded.

**Figure 1.**
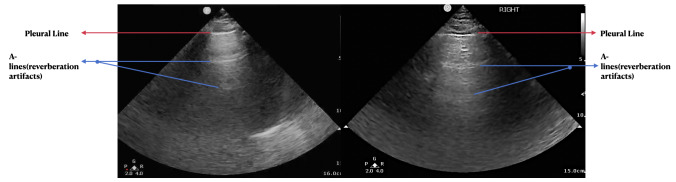
Bilateral predominant A-line pattern seen as a hyper-echoic line at the top of the image representing the pleural line (red arrow) and parallel hyper-echoic lines equidistant from the pleural line called reverberation artifacts (blue arrows).

**Figure 2.**
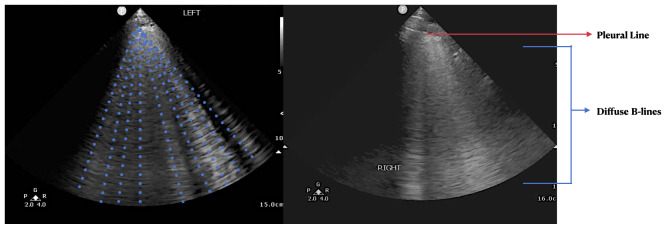
Bilateral predominant B-line pattern seen as a hyper-echoic lines (dotted blue lines on the left image and blue arrows on the right image) starting from the pleural line at the top of the image (red arrow) and extending vertically downwards towards the bottom of the screen without fading.

**Figure 3.**
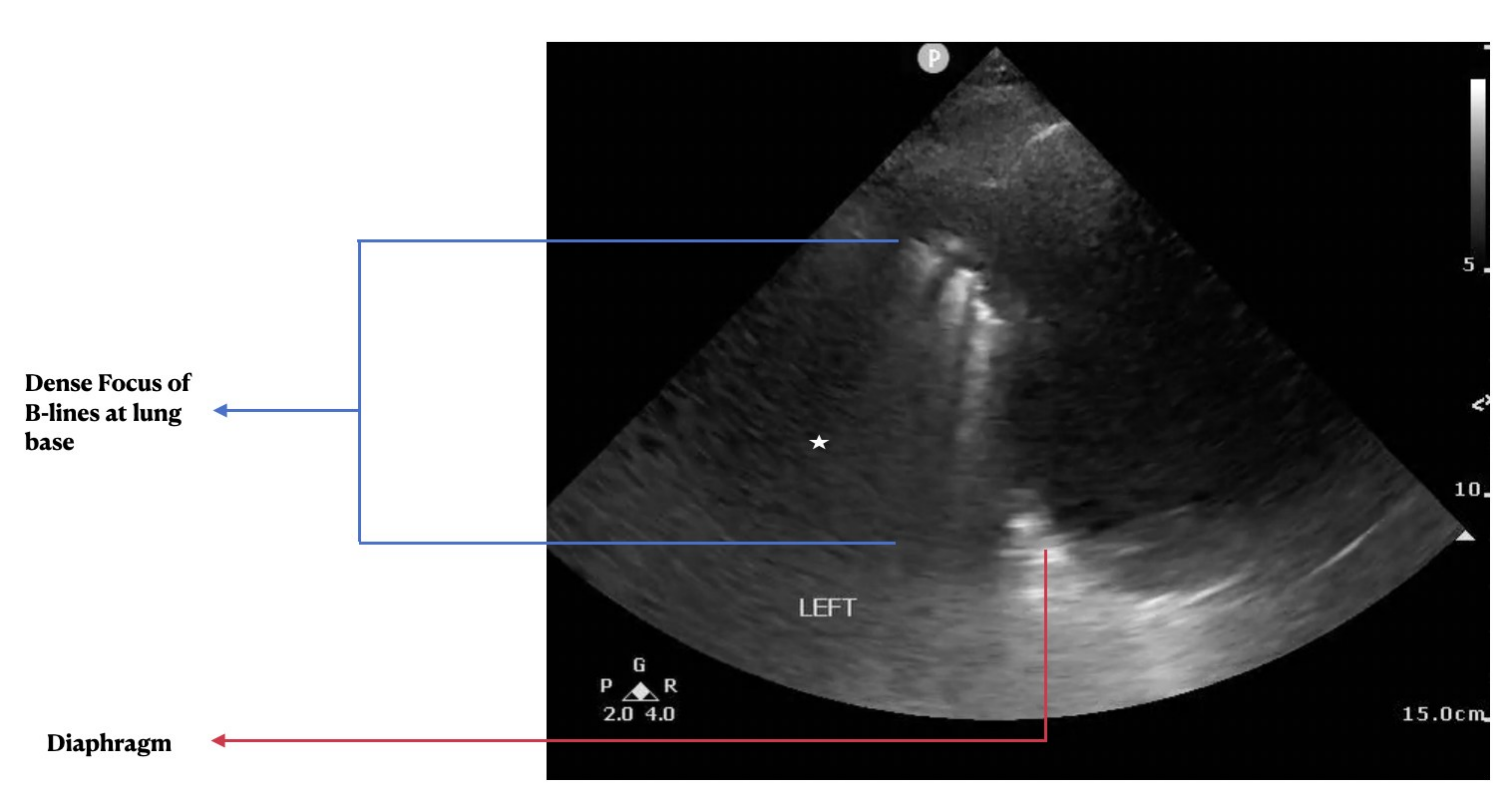
Cluster of B-lines seen as hyper-echoic lines (blue arrows) at the base of the lung identified by the diaphragm (red arrow) with the underlying spleen (white stars).

**Figure 4.**
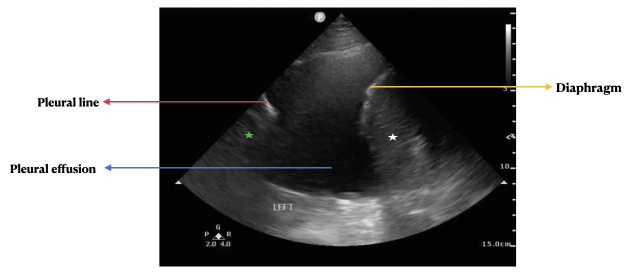
Pleural effusion seen as a hypo-echoic space (blue arrow) flanked by the diaphragm (yellow arrow) with the underlying spleen (white star) and the pleural line (red arrow) with the underlying lung parenchyma (green star).

History, physical exam, laboratory, radiological studies, final diagnoses and management decisions were reviewed. We identified three main lung POCUS patterns. These included lung sliding with bilateral A-lines and 0-2 B-lines per lung zone (normal lung POCUS, Figure 1), >2 B-lines bilaterally (predominant B-line pattern, Figure 2) and a predominant A-line pattern with focal, dense B-lines (Figure 3). In addition, patients with unilateral or bilateral pleural effusions (Figure 4) with any of the above patterns were also recorded. Lung POCUS findings were compared with CXRs performed within a 72-hour period, with CT scans, and cardiology-reviewed echocardiograms completed within one month of the lung POCUS examination.

### Statistical analysis

Summary statistics are reported as means and standard deviations for normally distributed variables, median values and ranges for non-normally distributed continuous variables, and as percentages and counts for categorical variables.

## Results

### Patient characteristics

From January 2023 to October 2023, lung POCUS exams were performed on 100 patients, consisting of 64% females and 36% males with a mean age of 57.26 +/-17.07 years. Of these patients, 36% were new consults and 64% were follow-up patients.

Among the 100 patients evaluated (Table 1), 49% had a history of smoking, 37% had asthma or COPD, 12% had interstitial lung disease, 6% had pleural effusions, and 5% had pulmonary hypertension. There was no pre-existing lung disease in 21% of patients. The most common presenting complaints were shortness of breath (63%), cough (32%), chest pain (14%), and wheezing (7%) (Table 1).

**Table 1.** Patient characteristics.

Age (mean +/− SD years)	57.26 +/− 17.07
Gender	N (%)
Female	64 (64)
Male	36 (36)
Co-morbidities	N (%)
History of smoking	49 (49)
Asthma/Chronic obstructive pulmonary disease (COPD)	37 (37)
Gastroesophageal reflux disease	14 (14)
Interstitial lung disease	12 (12)
Pleural effusion	6 (6)
Pulmonary hypertension	5 (5)
End-stage renal disease	4 (4)
Presenting complaint	N (%)
Shortness of breath	63 (63%)
Cough	32 (32%)
Chest pain	14 (14%)
Other	11 (11%)
Wheezing	7 (7%)
Fatigue	6 (6%)
Pleural effusion	6 (%)
Pre-op evaluation	6 (%)

Other- sleep related issues, night sweats, referrals from other providers, lightheadedness, weight loss, palpitations, hypoxemia, malaise, hypotension, gastroesophageal reflux disease, globus sensation, sleep disturbances, back pain.

### Lung point of care ultrasound findings

Seventy percent of lung POCUS exams were performed by residents rotating through the pulmonary outpatient clinic, 11% by pulmonary fellows, and 18% by pulmonary attendings. The median time to perform each lung POCUS and limited echocardiogram was 5 minutes (Q1: 4 minutes; Q3: 7 minutes; IQR: 3 minutes).

Seventy-one percent of the patients had a normal lung POCUS pattern (lung sliding with bilateral A-lines and 0-2 B-lines per lung zone, Figure 1). Bilateral B-lines (predominant B-line pattern, Figure 2) were seen in 13% of the patients and a predominant A-line pattern with focal dense B-lines (Figure 3) was seen in 10% of the patients. Pleural effusions (Figure 4) were demonstrated in 18% of the patients.

The most common diagnosis associated with a normal lung POCUS (Table 2) were obstructive airway diseases (31, 44%), obstructive sleep apnea (13, 18%), post-acute syndrome of COVID (7, 10%), cough due to gastro-esophageal reflux disease (9, 13%), and upper airway cough syndrome (7, 10%). The most common diagnoses associated with a bilateral B-line pattern were interstitial lung disease (7, 54%) and heart failure/end-stage renal disease (8, 62%). Focal dense B-lines were seen with atelectasis (3, 30%), abnormal CT findings including ground glass opacities, focal interstitial abnormalities and pulmonary nodules (3, 30%), and metastatic pulmonary lesions (2, 20%).

**Table 2.** Lung point of care ultrasound (POCUS) findings.

Lung POCUS findings	N (%-out of 100 patients)	Diagnosis	N	% (out of number of patients with a particular POCUS pattern)
Normal lung POCUS (bilateral A-line pattern with<3 or no B-lines)	71 (71%)	Obstructive airway disease	31	44
		Obstructive sleep apnea	13	18
		Miscellaneous	12	17
		Cough due to gastroesophageal reflux disease	9	13
		Post acute syndrome of COVID	7	10
		Upper airway cough syndrome	7	10
		CT abnormalities	5	7
		Shortness of breath of unclear etiology	4	6
		Venous thromboembolism	3	4
		Pulmonary hypertension	2	3
		Resolution of pneumonia	1	1
		Pneumothorax	1	1
Abnormal lung POCUS patterns	41 (41%)			
Bilateral B-lines	13 (13%)	Interstitial lung disease	7	54
		Heart failure/End-stage renal disease	8	62
Predominant A- lines with unilateral/Focal dense B-lines in a specific region	10 (10%)	Atelectasis	3	30
		CT abnormalities	3	30
		Pulmonary metastatic lesions	2	20
		Obstructive sleep apnea with deconditioning	1	10
		Lung implant	1	10
Pleural effusion	18 (18%)	Re- accumulation/New	8	44
		Resolving	7	39
		Stable/Unchanged	3	17

Miscellaneous - anxiety, obesity, deconditioning, sleep related disorders, anatomic chest wall deformity, post viral broncho- reactivity, anemia, medication effect - e.g. beta-blocker influence on heart rate, postural orthostatic tachycardia syndrome, evaluation of hypotension revealing endocrine disturbances and dehydration. CT abnormalities associated with a normal lung POCUS - resolving ground glass opacities, resolving lesions of inflammatory type interstitial lung diseases, pulmonary nodules. CT abnormalities associated with focal B-lines on lung POCUS - non-necrotizing granulomas, lesion of a localized interstitial lung disease, pulmonary nodules.

Lung POCUS was used to evaluate pleural effusions in 18% of patients. Of these cases, 8 (44%) revealed re-accumulation or new effusions, 7 (39%) had a resolving pleural effusion and 3 (17%) demonstrated stability (Table 2).

### Impact on management decisions

No additional studies were ordered in 51% of patients. Of the 49% who received additional testing, CT scans were ordered in 37%, CXRs in 10%, and echocardiograms in 17%. Pulmonary function tests or methacholine challenge tests were ordered in 73% of patients. Polysomnograms/home sleep tests were ordered in 10%, six- minute walk tests in 5%, sputum cultures in 2%, and bronchoscopies or right heart catheterizations were scheduled in 1%. Blood tests were ordered in 44%. Referrals were placed to cardiology (9%), sleep (6%), ear, nose and throat (5%), thoracic surgery and interventional pulmonology (4%), and allergy (4%). Of the 49% patients for whom follow-up imaging was performed, POCUS demonstrated concordance with CXR in 100% cases.

Table 3 highlights five anecdotal cases in which POCUS use guided provider decisions. When evaluating shortness of breath in two patients with known heart failure, POCUS was useful in showing improvement in or persistence of bilateral B-lines which impacted decisions about diuretic dosage. Two cases of pleural effusions are also highlighted. In the first case, POCUS showed worsening size of effusion compared to prior chest CT in a patient with metastatic colon cancer, which led to a thoracentesis that confirmed the diagnosis of a malignant pleural effusion. In the second case, a patient with known breast cancer and known malignant pleural effusion presented with shortness of breath. POCUS showed minimal effusion and therefore alternate causes of dyspnea were evaluated, as described in Table 3. Lung POCUS was also useful in ruling out pneumothorax in a post-discharge follow-up.

**Table 3.** Selected cases of management changes based on lung point of care ultrasound (POCUS) findings.

Chief complaint	Relevant past medical history	Relevant POCUS findings	Management/Conclusion	Follow-up chest imaging performed after clinic visit
Dyspnea on exertion	Heart failure	Bilateral lung sliding, A-lines without pleural effusions	Normal POCUS with evidence of obstruction on spirometry-inhaler prescribed with improvement in symptoms.	No
Follow up of pleural effusion	Colon cancer with lung metastasis	Moderate to large anechoic left pleural effusion, increased in size compared to prior CT chest	Concern for malignant pleural effusion - thoracentesis done, fluid studies confirmed diagnosis.	No
Shortness of breath	Breast cancer complicated by a malignant pleural effusion which was previously drained	Minimal right sided pleural effusion	No further pleural intervention was indicated.	No
Post discharge follow-up of pneumothorax	Recent admission for spontaneous pneumothorax that was managed conservatively	Bilateral lung sliding	Resolution of pneumothorax - Non-urgent CT chest ordered.	CT chest 6 weeks later confirmed resolution of pneumothorax
Post discharge follow-up of chronic heart failure exacerbation	Heart failure, chronic kidney disease, liver cirrhosis	B-lines bilaterally with no pleural effusions	Suspected heart failure exacerbation - increase in diuretic dose with improvement in shortness of breath.	No

## Discussion

To the best of our knowledge, literature on the role of lung POCUS in the pulmonary outpatient setting is scarce. Our primary objective was to describe the current practice and feasibility of POCUS use among physicians in our pulmonary outpatient practice.

Time constraints are a commonly reported barrier to POCUS use [8–11]. However, we demonstrated that the median time for lung POCUS was only 5 minutes. Other studies have also shown that a combined heart, lung, and vascular ultrasound performed in an outpatient setting was 11 minutes and that the total visit time was not significantly different between those undergoing a POCUS exam during their clinic encounter and those not [7]. Another barrier to POCUS use in a pulmonary outpatient clinic might be the funding and cost to acquire the machine. To support other institutions in justifying the acquisition and use of lung POCUS in pulmonary clinics, we recommend further exploring its potential billing and diagnostic role through prospective studies.

In selected cases, lung POCUS may have influenced decision-making. Of our patients, 71% of patients had a normal lung POCUS with a predominant A-line pattern. When subsequent imaging was performed, 100% of these normal lung POCUS studies were concordant with CXR or chest CT. Other studies have also not found any difference between the diagnoses made by CXR and lung POCUS and have shown that CXR does not provide any additional actionable information when compared to POCUS [5,13]. Therefore, a normal lung POCUS study may potentially replace CXR, which can sometimes be of lesser diagnostic value. In addition, lung POCUS provides more immediate actionable information by eliminating the longer wait times required to schedule, perform, and read outpatient chest radiography. There may be a cost-saving benefit when obtaining POCUS exams in clinic and foregoing CXR. Some in-patient studies have shown lower total and per-day hospital and radiology costs in admitted patients who had a lung POCUS exam, including a $743 reduction in patient cost for every lung POCUS exam performed [14,15].

Two conditions in which lung POCUS has been shown to be superior to CXR are pneumothorax and pleural effusions. Of the 18 pleural effusion cases evaluated, follow-up imaging was ordered for only 7 cases and there was a 100% concordance between POCUS findings and the CXR/CT scan. Several studies have demonstrated high negative predictive values of >98% and >94% for pneumothorax and pleural effusion in patients with dyspnea, respectively [16–18]. Lung POCUS has also been shown to have 100% sensitivity and specificity for pneumothorax and >95% sensitivity and specificity for pleural effusions in comparison to CXRs [5,19]. Real-time evaluation of pleural effusions with lung POCUS leads to an immediate diagnosis and can shorten the time to further management. Future studies are needed to evaluate the impact on time to intervention or further management.

We identified 7 patients with interstitial lung disease who exhibited a B-line pattern on lung POCUS. Although these patients were not followed longitudinally for this study, the findings suggest that lung POCUS may have the potential for monitoring disease progression in interstitial lung disease, and potential to reduce reliance on high-resolution CT, which is associated with significant radiation exposure [20].

Previous studies have demonstrated a strong positive correlation between the number and distance of B-lines in a given lung zone and CT-guided Warrick score for pulmonary fibrosis [21–23]. As well, Buda et al. developed an Ultrasound Fibrotic Index. This quantifies fibrosis based on pleural line irregularities and parenchymal artifacts, including the number of B-lines. This index shows association with the extent and severity of fibrosis as described by the Warrick model on CT [21,24].

Our present study has limitations. We performed an observational study as part of a QI effort; hence our aim was to evaluate the clinic workflow and environment for lung POCUS use, including the clinical decisions made as a result. However, we did not appraise the diagnostic accuracy and impact on management decisions. We did not explore the reasons behind why follow-up studies were ordered in certain cases and not in others. We also did not study whether lung POCUS changed presumed diagnoses before and after its use. Therefore, any conclusions we make here regarding the influence of lung POCUS on clinical decision making are hypothetical and not definitive. Since we did not include a comparator group (i.e., patients who did not receive POCUS), any conclusions around time savings or diagnostic value should be considered preliminary. This was a single center study with resident and fellow trainees, which may limit generalization to private practice settings. A cultural change is needed to increase POCUS acceptance in pulmonary outpatient clinics and may be achieved with more prospective clinic-centered studies that evaluate the role of POCUS in clinical management compared to conventional imaging.

## Conclusions

Our study suggested that lung POCUS is feasible in the pulmonary outpatient clinic and may have strong concordance with CXR in the evaluation of certain pulmonary conditions. Further studies are needed to compare lung POCUS findings with conventional imaging and to prospectively quantify its impact on the clinical management of patients. In addition, further investigation regarding providers' views on the role of lung POCUS in the outpatient setting and identifying ways to facilitate its incorporation in the clinical encounter can increase its use. Lastly, the amount of radiation, cost, and time to diagnosis saved by lung POCUS in the management of patients with pulmonary conditions should be further studied, as this may enable the provision of more affordable and timely care while limiting unnecessary adverse effects.
